# 

**Published:** 1913-04-12

**Authors:** 


### DOCTORS' WILLS.

Dr. Peter Redfern, M.D., F.R.C.S., D.Sc., formerly Regius Professor of Anatomy and Physiology at Queen's College, Belfast, has left personal estate in the United Kingdom valued at £96,279.

Dr. Alfred Baynard Duffin, M.D., F.R.C.P., F.R.C.S., successively Professor of Pathological Anatomy, Clinical Medicine, and Medicine at King's College, London, and Physician to the Church Missionary Society, has left net personalty £48,268.

### Buildings Contemplated and in Progress.

Arbroath.—Abroath Local Authority has had plans prepared for a smallpox hospital adjoining the epidemic hospital at Little Cairnie.

Bromley (Kent).—For alterations and additions to the isolation hospital for Beckenham and Bromley Joint Board a tender of £4,200 has been accepted.

Conisborough.—Doncaster and Mexborough Joint Hospital Board have approved an amended scheme for the extension of the isolation hospital to cost £5,626.

Dalkeith.—Mr. A. Murray Hardie, of Edinburgh, is architect for the new isolation hospital at Whitehill, which cost £5,000, and has recently been formally opened.

Dunfermline.—Plans submitted by Dr. G. Robertson for alterations to a house in East Port Street have been passed by the Dean of Guild Court. It is intended to effect alterations on the present structure and to erect a new surgical nursing home in the rear.

Eastbourne.—Mr. W. Chapman Field, borough architect, has prepared plans, etc., in connection with the infectious diseases hospital. The Local Government Board has held an inquiry into an application for sanction to a loan.

Epsom.—For the erection of the eleventh asylum on the Horton Estate, Epsom, for London County Council a tender of £335,577 has been accepted.

Gorleston.—Yarmouth Guardians have received sanction to borrow £2,678 for extensions to the children's homes.

Grimsby.—Mr. H. C. Scaping, Court Chambers, Grimsby, is architect for a children's home at Brighowgate. Tenders are invited for the works.

Huddersfield.—The Corporation General Purposes Committee has issued instructions for preparation of a scheme showing how Royds Wood Estate can be utilised for the provision of a sanatorium and a separate institution for children, an open-air swimming bath and open-air school, and recreation grounds.

Ladysbridge.—Banff Lunacy Board have adopted plans prepared by Mr. W. Kelly for extensions to Ladysbridge Asylum.

Leicester.—Messrs. Everard, Son, and Pick, 6 Millstone Lane, Leicester, are architects for new laundry, bath-house, matron's block, etc., at the Mental Hospital, Humberstone. A tender of £10,350 has been accepted.

Lincoln.—The Guardians have agreed to purchase land on Burton Road as a site for a new infirmary, to cost £25,000.

London.—A tender of £4,796 is recommended for acceptance by Hackney Guardians for a mortuary, workshops, etc.

London.—The Managers of the Metropolitan Asylum District have adopted the scheme prepared by Mr. T. W. Aldwinckle, F.R.I.B.A., for the enlargement of Tooting Bec Mental Hospital.

Manchester.—A crèche ward is proposed at Monsall Hospital for the Manchester Sanitary Committee. Particulars for tender may be obtained from the City Architect, Town Hall, on deposit of 21s.

Northfleet.—The Urban District Council has decided to apply for sanction to borrow £8,000 for the acquisition of Brook Vale Farm as a site for a smallpox hospital.

Oldham.—In connection with Oldham Royal Infirmary it is proposed to erect a convalescent home.

Orrell.—Lancashire Insurance Committee have submitted plans to the Local Government Board for a hospital to accommodate about sixty patients.

Orsett.—Alterations and additions are projected at Orsett Union Workhouse from particulars prepared by Mr. C. M. Shiner, A.R.I.B.A., 7 Adam Street, Adelphi, W.C. Tenders are under consideration.

Prescot.—Application is being made to the Local Government Board for sanction to a loan of £5,110 for the purchase of Eccleston Hall Estate for a tuberculosis hospital.

Rugby.—Mr. T. W. Willard, Market Place, Rugby, is architect for children's homes, laundry, etc., at Rugby Workhouse. Tenders are invited.

Sheffield.—The projected extensions to Sheffield Union Hospital are estimated to cost £35,937.

Sheffield.—The site for an Edward Memorial Cripples' Home has been presented by the Duke of Norfolk, and is situate in Rivelin Valley. Sketch plans by Mr. A. W. Kenyon, A.R.I.B.A., of George Street, Sheffield, have been forwarded to the School Management Committee for consideration.

Stafford.—A new ward block is to be erected at the Coton Field Asylum by Stafford Corporation. Particulars for tender may be obtained from Mr. W. Plant, A.M.I.C.E., Borough Hall, Stafford.

Staverton (Daventry).—Mr. J. B. Williams, Moot Hall, Daventry, is architect for an infectious diseases hospital at Staverton for the Rural Council. Tenders for the works are now under consideration.

Swinton, Lancs.—Mr. A. J. Murgatroyd, 23 Strutt Street, Manchester, is architect for reception wards at the Manchester Guardians' Schools at Swinton. Tenders are under consideration.

Ulverston.—Plans have been submitted to the Local Government Board by Lancashire Insurance Committee for a new hospital to accommodate about sixty patients.

### THE DRAMA OF "THE BOOK OF JOB."

Princess Christian, the President of the Ladies' Association of the Royal Hospital for Incurables, Putney Heath, has promised to attend a performance of the drama of "The Book of Job," by the Norwich Players, arranged by the Hon. Sybil Amherst. The performance has been unavoidably postponed, and will now be performed on Thursday, May 8, at the King's Hall, King's Street, Covent Garden, at 3 p.m. The entire proceeds will go towards the funds of the Royal Hospital for Incurables, Putney Heath. Tickets, £2 2s., £1 1s., 10s. 6d., and 5s., can be obtained from The Lady Loch, 51 Lennox Gardens, and Miss Allcroft, Hon. Secretary, 93 Elizabeth Street, Eaton Square, S.W.

### Gifts and Bequests.

Sir John Ramsden has sent £1,000 to Mr. Austen Chamberlain's Fund for the Extension of the London School of Tropical Medicine.

Mr. E. S. Wills, of Ramsbury Manor, has cleared off the debt of £300, which was reported at a meeting of the Savernake Hospital, Marlborough.

Mr. W. C. Bridgeman, M.P., the hon. treasurer of the Florence Nightingale Hospital for Gentlewomen, 19 Lisson Grove, N.W., has received from an anonymous friend a promise of £100 towards the extension of the hospital, on condition that four sums of a like amount are also given.

The Rev. George Campbell Williams, D.D., of Dublin, who served as an Army chaplain in the Baltic Expedition of 1855 and afterwards as chaplain to the Lord Lieutenant of Ireland and principal chaplain to the Forces at Dublin, left personal estate valued at £13,031. Subject to his wife's life interest, he left £500 to the Protestant Home for the Dying, Dublin.

Miss Mary Augusta Scott, of South Hampstead, has left £1,000 to the Royal Free Hospital, Gray's Inn Road, to found a bed "in loving memory of Emma Brandon" to bear her name; and, subject to one life interest, she has left £1,000 to the Northampton General Hospital, in memory of her parents, William and Sophia Scott, and £1,000 to the Middlesex Hospital to endow a bed in the cancer ward bearing her own name.

The late Mr. John Jones, of Wrexham, a generous donor to churches and hospitals, recently bought Roseneath for the Wrexham Infirmary and Claremont Hydro, Rhyl, as a convalescent home for the inhabitants of Wrexham.

### Appointments.

John D. Comrie, M.D., F.R.C.P., has been appointed an assistant physician to the Edinburgh Royal Infirmary.

Mr. Cecil Rowntree, M.B., B.S., F.R.C.S., has been appointed surgeon to the St. Marylebone General Dispensary.

The executive committee of the Edinburgh Hospital for Women and Children has confirmed the appointment of Dr. Janet Mowat as dispensary medical officer in place of Mrs. Barnetson, M.B., Ch.B., who has resigned.

Mr. Ivy Mackenzie, B.Sc., M.D., has been appointed visiting physician to the Glasgow Victoria Infirmary, in succession to Dr. John Lowe, who has resigned. A graduate of Glasgow University, Dr. Mackenzie has been for the past three years a director of the Western Asylums Research Institute.

The following appointments have been made at the Hampstead General and North-West London Hospital:—House physician (Hampstead): Mr. A. Bloom, M.B., Ch.B. (Edin.); house surgeon (Hampstead): Mr. James Erlank, M.B., Ch.B. (Edin.); assistant casualty officer (Camden Town): Mr. J. M. Wallace, M.A. (Cantab.), M.R.C.S., L.R.C.P.

### Obituary.

Dr. William Robinson Evans, of Dublin, died suddenly at a meeting on Monday. The announcement of his death to his aged sister proved too much for her weak health, and she died soon after hearing the news.

The Rev. Henry Spurrier, rector of Roughton with Haltham, Lincolnshire, died last week, aged eighty-three. He was appointed to the rectorship of Roughton with Haltham in 1867. He was one of the governors of Woodhall Spa Hospital, on which we published a report on December 16, 1911.

Mr. Arnold Rogers, formerly dean of the Dental Hospital, has died, aged eighty-eight. He took his M.R.C.S. in 1847, and was one of those who worked for the passing of the Dentists Act and helped to induce the Royal College of Surgeons to institute a diploma in dental surgery. He was at one time president of the Odontological Society.

The death took place last week, from bronchitis, of the Rev. W. H. Kerr, S.J., a grandson of the sixth Marquis of Lothian, who was early associated with the movement for founding the Nelson Hospital, Wimbledon. He was an active member of its council. Father Kerr's courtesy and kindliness will be much missed.

Dr. Samson Gemmell, Professor of the Practice of Medicine in Glasgow University, has died, aged sixty-five. Of Scottish birth and educated at Glasgow University, where he graduated M.D. in 1880, he became assistant to the late Sir William Gairdner, both at the Glasgow Royal Infirmary and when the latter was Professor at Anderson's College. Later Dr. Gemmell succeeded to the Chair of Clinical Medicine at Glasgow, and five years ago he was appointed to the Chair which he occupied till his death. He was also well known in private practice, and was a consultant to whom many medical men and patients were glad to resort.

Dr. Jordan Lloyd, F.R.C.S., M.D., a well-known Birmingham surgeon and senior surgeon at Queen's Hospital, has died, aged fifty-nine. Educated at the old Medical School of Queen's College and at Durham University, he joined the permanent staff of Queen's Hospital in 1883. As Professor of Surgery at Birmingham University, and consulting surgeon to several other institutions, his reputation as an operator was great. He was also Lieutenant-Colonel, R.A.M.C., Territorial Force, and commanded the 1st Southern General Hospital, South Midland Division. He had suffered from angina pectoris.

### Personalia.

Sir Savile Crossley left London a fortnight ago for Japan, and letters will not be forwarded to him. The governors of King Edward's Hospital Fund for London have appointed Mr. Frederick M. Fry to act as honorary secretary in his absence, and Mr. Fry has kindly consented to serve.

### "BOVRIL" PICTURES.

Regular readers of The Hospital are aware beyond the need of special reminder that the firm of "Bovril" has made a practice of organising a picture scheme. The pictures on the latest occasion, as usual, are of a kind certain to appeal to well-defined sections of the general public, and the fact that the "Bovril" picture scheme is still in force is the best testimony to the public's appreciation of the examples of pictorial art which are thus on a simple method placed within their reach.

